# Rampant Reticulation in a Rapid Radiation of Tropical Trees—Insights from *Inga* (Fabaceae)

**DOI:** 10.1093/sysbio/syaf027

**Published:** 2025-05-04

**Authors:** Rowan J Schley, Rosalía Piñeiro, James A Nicholls, Flávia Fonseca Pezzini, Audrey Farbos, Gwilym P Lewis, Jens J Ringelberg, Catherine Kidner, Alex D Twyford, Kyle G Dexter, R Toby Pennington

**Affiliations:** Department of Geography, University of Exeter, Laver Building, North Park Road, Exeter, Devon EX4 4QE, UK; Grupo de Investigación en Biología Evolutiva, CICA, Departamento de Biología, Campus A Zapateira sn., Universidade da Coruña, A Coruña 15071, Spain; Australian National Insect Collection, CSIRO, Clunies Ross Street, Canberra, ACT, 2601, Australia; Royal Botanic Garden Edinburgh, 20a Inverleith Row, Edinburgh EH3 5LR, UK; Royal Botanic Garden Edinburgh, 20a Inverleith Row, Edinburgh EH3 5LR, UK; University of Exeter Sequencing Service, Geoffrey Pope Building, Stocker Road, Exeter, Devon EX4 4QD, UK; Accelerated Taxonomy Department, Royal Botanic Gardens, Kew, Richmond, Surrey TW9 3AE, UK; School of Geosciences, Crew Building, King's Buildings, University of Edinburgh, Edinburgh EH9 3FFUK; Royal Botanic Garden Edinburgh, 20a Inverleith Row, Edinburgh EH3 5LR, UK; Institute of Molecular Plant Sciences, School of Biological Sciences, Daniel Rutherford Building, King's Buildings, University of Edinburgh, Edinburgh EH9 3BF, UK; Royal Botanic Garden Edinburgh, 20a Inverleith Row, Edinburgh EH3 5LR, UK; Institute of Ecology and Evolution, Ashworth Laboratories, King's Buildings, University of Edinburgh, Edinburgh EH9 3FL, UK; Royal Botanic Garden Edinburgh, 20a Inverleith Row, Edinburgh EH3 5LR, UK; School of Geosciences, Crew Building, King's Buildings, University of Edinburgh, Edinburgh EH9 3FFUK; Department of Geography, University of Exeter, Laver Building, North Park Road, Exeter, Devon EX4 4QE, UK; Royal Botanic Garden Edinburgh, 20a Inverleith Row, Edinburgh EH3 5LR, UK

**Keywords:** Amazon, diversification, hybridization, introgression, phylogenomics, radiation, rainforest, syngameon

## Abstract

Evolutionary radiations underlie much of the species diversity of life on Earth, particularly within the world’s most species-rich tree flora—that of the Amazon rainforest. Hybridization occurs in many radiations, with effects ranging from homogenization of divergent species to the generation of genetic and phenotypic novelty that fuels speciation. However, the influence of hybridization on Amazonian tree radiations has been little studied. We address this using the ubiquitous, species-rich, Neotropical tree genus *Inga*, which typifies rapid radiations of rainforest trees. We assess patterns of gene tree incongruence to ascertain whether hybridization was associated with rapid radiation in *Inga.* Given the importance of insect herbivory in structuring rainforest tree communities (and hence the potential for hybridization to promote adaptation through admixture of defense traits), we also test whether introgression of loci underlying chemical defenses against herbivory occurred during the radiation of *Inga.* Our phylogenomic analyses of 189/288 *Inga* species using >1300 target capture loci showed widespread introgression in *Inga*. Specifically, we found widespread phylogenetic incongruence explained by introgression, with phylogenetic networks recovering multiple introgression events across *Inga* and up to 20% of shared, likely introgressed, genetic variation between some species. In addition, most defense chemistry loci showed evidence of positive selection and marginally higher levels of introgression. Overall, our results suggest that introgression has occurred widely over the course of *Inga*’s history, possibly in a syngameon scenario, likely facilitated by extensive dispersal across Amazonia. Furthermore, in some cases, introgression of chemical defense loci may influence adaptation in *Inga*.

Rapid evolutionary radiations that generate exceptionally species-rich groups are a fundamental component of biodiversity (Hughes et al. 2015). Hybridization (interbreeding between species) is frequent in rapid evolutionary radiations (Seehausen 2004), but its evolutionary role has been long debated. While hybridization can result in “speciation reversal” that reduces diversity (Vonlanthen et al. 2012; Kearns et al. 2018), or may be “lineage-neutral” and have no effect on diversification (Justison et al. 2023), it is frequently invoked as a catalyst of rapid radiation (e.g., Barrier et al. 1999; Meier et al. 2017; Lamichhaney et al. 2018). This is because hybridization can “reshuffle” existing genetic variation, generating genomic and phenotypic novelty (Rieseberg et al. 2007; Marques et al. 2019) that may confer adaptation to new environments (e.g., adaptive introgression and/or transgressive segregation (Rieseberg et al. 1999; Suarez-Gonzalez et al. 2018)) or lead to re-sorting of intrinsic incompatibilities (e.g., Bateson–Dobzhansky–Muller incompatibilities) that promote reproductive isolation and hence rapid speciation (Schumer et al. 2015).

It is possible to detect genetic admixture and infer past reticulation events in a clade through examining gene tree conflict (Doyle 1992; Naciri and Linder 2015). The proportions of different conflicting gene tree topologies can indicate the relative contributions of introgression (transfer of genetic material following persistent hybridization) and incomplete lineage sorting (ILS) to phylogenetic incongruence (e.g., Green et al. 2010; Durand et al. 2011; Pease et al. 2018). This incongruence can also help estimate the relative genetic contributions of progenitor lineages to introgressant descendants (Patterson et al. 2012). There is a growing body of work that explores incongruence to better understand introgression across the tree of life, particularly in plants (e.g., in oaks McVay et al. 2017 and willows Wagner et al. 2020), but only recently have such studies been undertaken in the most species-rich flora on Earth—that of Neotropical rainforests (Schley et al. 2020; Larson et al. 2021; reviewed in Schley et al. 2022).

The flora of Neotropical rainforests is remarkable in its species diversity (Antonelli and Sanmartín 2011; Ulloa Ulloa et al. 2017; Raven et al. 2020), particularly at local scales—there are more tree species in a single hectare of the Amazon (ca. 655 spp. (Valencia et al. 2004)) than in all of Europe (ca. 454 spp. (Rivers et al. 2019)). Many species-rich Neotropical plant groups arose through recent rapid radiations (e.g., Erkens et al. 2007: Annonaceae; Koenen et al. 2015: Meliaceae), but the influence of hybridization on tropical plant radiations has been little studied, and virtually not at all in tropical rainforest trees (reviewed in Abbott (2017)). The prevailing view, based largely on morphological patterns, has been that hybrids between rainforest tree species are exceptionally rare (e.g., Ashton 1969), but this is challenged by recent genomic data for a few Amazonian tree species (e.g., *Brownea* (Schley et al. 2020); *Eschweilera* (Larson et al. 2021)).

The tree genus *Inga* Mill. (Fabaceae) is widespread and abundant in Neotropical rainforests, and was the first documented example of rapid radiation in the Amazonian tree flora (Richardson et al. 2001). *Inga* exhibits the highest diversification rate of any Amazonian tree genus (Baker et al. 2014), with ca. 300 species arising in the last ca. 10 Ma (Ringelberg et al. 2023). Similar recent, rapid radiation events in other tree genera gave rise to a large portion of the Amazonian tree flora—over half of Amazonian tree species belong to genera with >100 species (Dexter and Chave 2016). *Inga* is an ideal study system to understand the influence of hybridization on rapid rainforest tree radiations due to the large volume of previous work examining the diversification, ecology, and biogeography of the group (Richardson et al. 2001; Kursar et al. 2009; Dexter et al. 2017; Forrister et al. 2019) coupled with its ubiquity and species diversity in the Amazon (Pennington T. D. 1997). Previous phylogenetic work on *Inga*, based on Sanger sequencing of relatively few species, revealed low resolution of species-level relationships (Richardson et al. 2001; Kursar et al. 2009; Dexter et al. 2010), with resolution improving in later phylogenomic studies that sequenced 264 loci for 22 *Inga* species (Nicholls et al. 2015). Here, we generate the most comprehensive phylogenetic tree of *Inga* to date, comprising >1300 loci and 189 species, greatly improving resolution of *Inga* species relationships to help understand whether hybridization influenced diversification.

Hybridization may be more widespread than initially thought in rainforest tree radiations like *Inga*, first and foremost because of their remarkable level of co-occurrence in local rainforest communities. Up to 19 species of *Inga* can coexist in 1 ha, and such high local diversity is typical of many other species-rich Amazonian tree genera (e.g., *Protium* and *Eschweilera* (Valencia et al. 1994; Larson et al. 2021)), some of which have emerging evidence of hybridization (e.g., between 3 *Eschweilera* species in Manaus, Brazil (Larson et al. 2021)). Furthermore, there is substantial overlap in flowering times of many *Inga* species, which share a wide range of pollinators due to their generalist pollination syndrome (Koptur 1983), and recent work using microsatellites suggested hybridization occurs between 2 *Inga* species in Peru (Rollo et al. 2016).

These dispersal-assembled local communities of *Inga* (Dexter et al. 2017) are largely structured by insect herbivore pressure, such that co-occurring *Inga* species differ more in their chemical defenses against herbivores than expected by chance (Kursar et al. 2009; Endara et al. 2022). This is because higher densities of conspecifics with the same defenses leads to increased mortality from herbivores that can overcome these defenses (Janzen 1970; Connell 1971; Forrister et al. 2019). Divergent adaptation in chemical defense traits is documented in *Inga* over evolutionary timescales (Forrister et al. 2023), likely driven by these negative density-dependent processes over ecological timescales (Forrister et al. 2019), as selection favors defenses that local herbivores cannot overcome. This suggests that it is adaptive to possess rare defense chemistry phenotypes. Therefore, the transfer of defense chemistry loci between *Inga* species via hybridization, followed by positive selection on those loci, may be predicted to facilitate colonization of new ecological space and eventually lead to speciation. To our knowledge, this has never been investigated from a genomic perspective. Therefore, here we aim to:

1. Infer the diversification history of *Inga* by generating the most comprehensive phylogenetic tree of the genus to date;
2. Assess patterns of phylogenetic incongruence and reticulate evolution, resulting from hybridization, across *Inga*’s evolutionary history;
3. Assess whether hybridization may have contributed to *Inga*’s rapid diversification through transfer of adaptive genomic loci underlying chemical defense against herbivores.

## Materials and Methods

### Taxon Sampling and DNA Sequencing

We performed phylogenomic analyses on target capture sequencing data from 189 of the 282 accepted *Inga* species (67%), 63 of which were sequenced for this study, and 126 of which were taken from previous work (one from Nicholls et al. 2015; 79 from Ringelberg et al. 2023; 46 from Nicholls et al. 2025). Our sampling was based on a list of all accepted *Inga* species compiled using the World Checklist of Vascular Plants (WCVP 2020) (as of June 2021) and a monograph of *Inga* (Pennington T. D. 1997). Preliminary analyses identified 13 subclades within *Inga*, with the size of these used to ensure proportional per-subclade downsampling for computationally intensive downstream analyses.

To contextualize our *Inga* analyses and gain a better understanding of reticulation histories at broader phylogenetic scales, we collated sequence data from many outgroup species generated by previous studies (Nicholls et al. 2015; Koenen et al. 2020; Ringelberg et al. 2022). We included all 9 genera and 32 other species from the “Inga clade” (*sensu*  Koenen et al. 2020) within which *Inga* is nested, including its sister genera *Zygia*, *Macrosamanea, Ingopsis*, and *Pseudocojoba* (Ferm and Rydin 2024). In addition, we included a further 73 species comprising all 42 genera from the “Ingoid clade” (*sensu*  Koenen et al. 2020) within which the Inga clade is nested. Finally, we included 22 species from the broader Mimosoid legume clade, giving a total of 127 outgroup species. A list of accessions sampled including species, sampling location, data source, and voucher information is detailed in Supplementary Table S1.

DNA was extracted from 20 mg of dried leaf material with the DNeasy Plant Mini Kit (Qiagen, Hilden, Germany) using modifications described in Nicholls et al. (2015). DNA library preparation, enrichment, and sequencing were carried out either by Arbor BioSciences (Ann Arbor, MI, USA) or the University of Exeter sequencing service (Exeter, UK) following the NEBnext Ultra II FS protocol (New England Biolabs, Ipswich, MA, USA). Targeted bait capture was performed with a subfamily-specific “Mimobaits” bait set (Nicholls et al. 2015; Koenen et al. 2020) using the MyBaits protocol v.2 and 3 (Arbor Biosciences). The Mimobaits set targets 1320 loci, including 113 genes coding for enzymes underlying anti-herbivore defense chemistry in *Inga* (hereafter “defense chemistry” loci). Other loci targeted by the Mimobaits set are “single-copy phylogenetically informative” loci that were selected due to their high levels of informative substitutions and the fact they were single-copy (1044), “differentially expressed” loci that had different numbers of transcriptome reads between the species used to design the baits (109) and “miscellaneous” loci (54), which are un-annotated but contain phylogenetic signal. Within the 1044 “phylogenetically informative” genes, 943 were published in Koenen et al. (2020), while the 111 that remain unpublished were generated by Koenen et al. based on transcriptomes of *Inga* leaves and roots, using the ortholog detection protocol in Koenen et al. (2020)—see https://github.com/erikkoenen/mimobaits/. Enriched libraries were sequenced using the NovaSeq 6000 platform with a paired-end 150 bp run on 2 S1 flow cell lanes.

### Sequence Assembly, Trimming, and Alignment

All analyses were conducted on the UK Crop Diversity Bioinformatics HPC Resource. DNA sequencing reads were quality-checked with fastqc 0.11.3 (Andrews 2010) and were trimmed using Trimmomatic 0.3.6 (Bolger et al. 2014) to remove adapter sequences and to quality-filter reads. Trimmomatic settings permitted <2 mismatches, a palindrome clip threshold of 30, and a simple clip threshold of 10. Bases with a quality score <28 and reads shorter than 36 bases long were removed from the data set. Following quality-filtering, reads were mapped to target loci using bwa 0.7.17 (Li and Durbin 2009), loci were assembled using SPAdes 3.11.1 (Bankevich et al. 2012) with a coverage cutoff of 5× and exons were extracted with Exonerate (Slater and Birney 2005), all of which are implemented in the HybPiper pipeline 1.2 (Johnson et al. 2016). Recovery of loci was visualized using the “gene_recovery_heatmap.R” script distributed with HybPiper. To improve the signal:noise ratio and remove possible paralogs from our *Inga* data set, we used the Putative Paralogs Detection pipeline 1.0.1 (PPD; https://github.com/Bean061/putative_paralog; Zhou et al. (2022)), described in Supplementary Methods.

We conducted all subsequent analyses on 3 data subsets to include a broad range of phylogenetic scales, and to assess the influence of paralogy on our analyses. All data sets comprised a single accession per species, the first of which included 189 *Inga* species with *Zygia* sp. “*mediana*” as the outgroup (data set “Singlesp”). The second data set included 127 outgroup species and proportional per-subclade sampling of 50 *Inga* species, chosen to include the *Inga* species with the best locus recovery per subclade (data set “Outgroup”). The third and final data set comprised the same 189 accessions as the “Singlesp” *Inga* data set, but loci were assembled and cleaned using the PPD pipeline, with paralogous loci removed (data set “PPD”).

Targeted loci for each of the 3 data sets were aligned by gene region using 1000 iterations in mafft 7.453 (Katoh and Standley 2013) with the “*—adjustdirectionaccurately*” flag to incorporate reversed sequences. These alignments were then cleaned using the “-*automated1*” flag in trimAl 1.3 (Capella-Gutiérrez et al. 2009), and realigned with mafft using the “*—auto”* flag. This resulted in 1305 refined alignments for the “Singlesp” data set, 1044 for the “Outgroup” data set, and 1267 for the “PPD” data set. Alignment summaries detailing proportions of variable sites and missing data were then generated with amas (Borowiec 2016).

### Phylogenomic Analyses

Gene trees were inferred for each locus alignment across the 3 data sets using IQ-TREE (Nguyen et al. 2015) by selecting the best-fit substitution model (*-MFP*) while reducing the impact of severe model violations (*-bnni*) with 1000 ultrafast bootstrap replicates. Following this, a “species tree” was generated based on the best-scoring IQtrees using astralMP 5.15.5 under the default parameters (Zhang et al. 2018) for all 3 data sets. Following concatenation of all locus alignments within the “Singlesp” data subset using amas, we used the same parameters as above to infer a phylogenetic tree from the concatenated supermatrix using IQ-TREE. Finally, we visualized shared genetic variation among species by building neighbor net plots with uncorrected P-distances in SplitsTree v4.14.6 (Huson and Bryant 2005) for each of the 3 data sets.

### Analyzing Incongruence and Reticulation

To assess incongruence among our gene trees, we estimated 3 metrics implemented in the Quartet Sampling method 1.3.1 (https://www.github.com/fephyfofum/quartetsampling; Pease et al. (2018)) based on each data set’s astral species tree, using 100 replicate runs. For each node, we estimated Quartet Concordance (QC) to assess whether there was incongruence, where QC = 1 indicates the congruent topology is most frequent, QC = 0 indicates equal proportions of congruent and incongruent topologies, and QC = −1 indicates that incongruent topologies are most frequent. We also estimated Quartet Differential (QD) to assess whether one specific incongruent topology was favored, and Quartet Informativeness (QI) to assess whether data were sufficiently informative to distinguish well-supported incongruence from lack of signal (QI not shown as all data sets were informative).

Having assessed incongruence across our 3 data sets, we then visually investigated gene tree conflict of certain higher-level relationships highlighted in the Quartet Sampling analysis with DiscoVista (Sayyari et al. 2018) (subclades defined in Supplementary Table S2). We interpreted gene tree conflict as “low” if the proportion of gene trees supporting the species tree topology was >30% higher than for both alternatives, and nodes with one conflicting topology above the 33% “equal frequency” threshold were examined more closely as possibly suggesting introgression (after Kuhnhäuser et al. (2021)).

We then assessed whether the incongruence we inferred was caused by introgression or ILS using the *Dtrios* function in Dsuite (Malinsky et al. 2021). We used Patterson’s D-statistic (i.e., the “ABBA-BABA” test; Green et al. 2010; Durand et al. 2011) and estimated the proportion of shared variation between species using the *F*_4_ ratio (Patterson et al. 2012), where for both metrics values closer to 1 indicate more introgression. We generated an input VCF file for *Dtrios* for each of the 3 data sets by calling SNPs using bwa 0.7.17, samtools 1.13 (Danecek et al. 2021) and bcftools 1.13 (Li 2011) as in Joana Meier’s “Speciation Genomics” github (https://speciationgenomics.github.io), using our target bait set sequences as the reference. The resulting VCFs were filtered to contain sites with >8× coverage and a quality score >20, as well as removing taxa with >50% total missing data.

For each taxon trio test set, we additionally used the “*--abbaclustering*” tool in Dsuite to account for variation in substitution rate across clades, and so more accurately infer introgression without false positives resulting from homoplasy (Frankel and Ané 2023; Koppetsch et al. 2024). To further minimize the effect of substitution rate variation for the Outgroup data set, we only included Ingoid clade species in our Dsuite analysis, along with genera from its close sister group (*Jupunba/Hydrochorea/Punjuba/Pseudalbizia*), using *Cedrelinga/Pseudosamanea/Chloroleucon/Samanea/Boliviadendron/Enterolobium/Albizia* as outgroup. For the *Inga* Singlesp and PPD analyses, we used the closely related *Zygia* sp. “*mediana*” as outgroup. We assessed significance of each test using 20 block jackknife resampling runs (ca. 50,000–65,000 variants per block), following which we corrected *D* and ABBA-clustering *P-*values for multiple testing with the Benjamini–Hochberg correction in RSTATIX (Kassambara 2020) using R v 4.2.1 (R Core Team 2021) . Finally, we filtered out test sets without significant ABBA-clustering (indicating homoplasy), and visualized our D-statistic and *F*_4_ ratio estimates with Ruby scripts available from https://github.com/mmatschiner.

*D*-statistics and *F*_4_ ratios are best at detecting recent introgression (Bjorner et al. 2024), and older hybridization events can result in correlated *F*_4_ ratios between related species if introgressed variation is inherited by descendent lineages. We inferred deeper introgression events using *F*_branch_ (Malinsky et al. 2018) for each combination of taxa, filtered results to only include trios with significant ABBA-clustering, and plotted scores with the Dsuite “dtools.py” utility. *F*_branch_ is more conservative than other metrics (e.g., *F*_4_ ratio), and can place introgression events on internal branches of the phylogeny, because it calculates a median of *F*_4_ ratios between closely related branches.

To model historical reticulation across *Inga* and outgroup species, we inferred phylogenetic networks with SNaQ!, implemented in the Julia v1.7.2 (Bezanson et al. 2017) package PhyloNetworks v0.16.2 (Solís-Lemus et al. 2017). We inferred phylogenetic networks from representative downsampled subsets of our “Singlesp” and “Outgroup” data sets with between 0 and 4 reticulation events (*h*), due to computational limitations, excluding the PPD data set due to the minimal effect of paralogs on our analyses. We estimated networks by calculating quartet concordance factors (CF) for each node, which were also used to estimate γ values (probabilities of ancestral contribution to hybridization events). The best-fit network was chosen using negative log-pseudolikelihood comparison, selecting the *h-*value above which likelihood scores did not improve (*hmax*). We performed the same analysis on representative subsets of *Inga* subclades to better understand within-subclade reticulation. Downsampled data sets prioritized accessions with the highest locus recovery, aiming for proportional sampling of subclades. Details of *D*- and *F*-statistics, as well as accession selection for *PhyloNetworks*, are provided in Supplementary Methods.

### Assessing Per-Locus Introgression and Selection

We assessed whether defense chemistry loci experienced elevated introgression and selection relative to other loci in *Inga*. To do this, we first estimated the per-locus proportion of introgression using the *f*  _*dM*_ statistic (Malinsky et al. 2015) in Dsuite. *f*  _*dM*_ more accurately infers introgression in small genomic windows than *D-*statistics (Martin et al. 2014; Malinsky et al. 2015), while using the same sampling design (i.e., 3 taxa and an outgroup). We performed *f*  _*dM*_ analysis on 3 “subclade subsets,” which were selected based on inferred introgression events from PhyloNetworks and Dsuite. Each analysis was performed for all taxa together grouped by subclade, the first including all species from the Leiocalycina + Vulpina + Red hair subclades, the second between the Microcalyx grade + Leiocalycina + Redhair subclades, and the third between the Bourgonii + Microcalyx grade + Red Hair subclades (selected subclades shown in Supplementary Fig. S1; subclade selection described in Supplementary Methods). We used all non-“Fast clade” species as outgroups to minimize the effect of substitution rate variation on *f*  _*dM*_ estimates. For each subclade subset, we calculated *f*  _*dM*_ using nonoverlapping windows of 50 informative SNPs with a rolling mean of one window. We took the absolute values of all *f*  _*dM*_ scores, as we were only interested in comparing the magnitude of introgression across loci, and calculated a mean *f*  _*dM*_ score per-locus for downstream analyses.

We then assessed whether each of our target capture loci experienced positive selection (i.e., more nonsynonymous nucleotide changes than synonymous changes) on at least one branch and at least one site using BUSTED (Murrell et al. 2015). We prepared our target capture alignments for BUSTED analysis by trimming nonhomologous sequence fragments (i.e., intronic regions captured either side of exons), masking misaligned amino acid residues and producing codon-aware alignments using OMM_MACSE (Ranwez et al. 2011, 2021). Using these codon-aware alignments, we tested for the presence of selection in each locus across the same 3 subclade subsets of our *Inga* “Singlesp” data set for which *f*  _*dM*_ scores were calculated. We accounted for false positives by adjusting the selection test *P-*values output by BUSTED with a 5% FDR (false discovery rate) in R (Benjamini and Hochberg 1995). We assessed whether there were associations between locus selection result (“under selection” if the BUSTED FDR *P-*value < 0.05) and locus annotation (“defence chemistry,” “differentially expressed,” “single-copy phylogenetically informative,” and ‘miscellaneous’) using χ^2^ tests in R. We also visualized associations between selection result and locus annotation using the R package *corrplot* (Wei et al. 2017).

Finally, we used analysis of covariance (ANCOVA) in R to assess whether variation in our per-locus *f*  _*dM*_ estimates was explained by interactions between 3 variables. The variables were locus annotation, selection result, and the length of the locus alignment (to control for differences in the number of sites between loci). Response variables were square-root transformed to improve normalcy for all subsets except the Bourgonii + Microcalyx grade + Red hair subset, which was log-transformed. The heteroscedasticity of residuals was examined using the *plot()* function in R, and η^2^ (a measure of effect size that considers variance explained by both the independent variable and the covariate) was calculated with the R package *effectsize* (Ben-Shachar et al. 2020). Box plots of per-locus *f*  _dM_ estimates were generated using *ggplot2* (Wickham 2016) in R, with locus annotation and selection result as grouping variables. We performed both *f*  _*dM*_ and BUSTED analyses on all 1305 refined loci for each data subset, but several loci were filtered out both by BUSTED and *f*  _*dM*_, and so we retained only those that were present in both analyses.

## Results

### Phylogenomic Analyses

We achieved a mean of 72.05% reference length recovery onto the *Mimobaits* bait sequences (78.99%, excluding outgroups, across 1305 loci). Overall, the “Singlesp” data set had 1.53 × 10^6^ sites, with a mean of 0.31% variable sites and 2.30% missing data across all loci. The PPD data set had 1.89 × 10^6^ sites, with a mean of 0.30% variable sites and 3.02% missing data. Finally, the Outgroup data set had 1.44 × 10^6^ sites, with a mean of 0.66% variable sites and 12.45% missing data. The “Outgroup” data set comprised only of the 1044 “single-copy phylogenetically informative” loci sequenced by previous studies (Koenen et al. 2020; Ringelberg et al. 2022). Heatmaps showing percentage recovery per locus are available in Supplementary Fig. S2, along with summaries of sites, variability, and missing data per locus in Supplementary Table S3.

**Figure 1.**
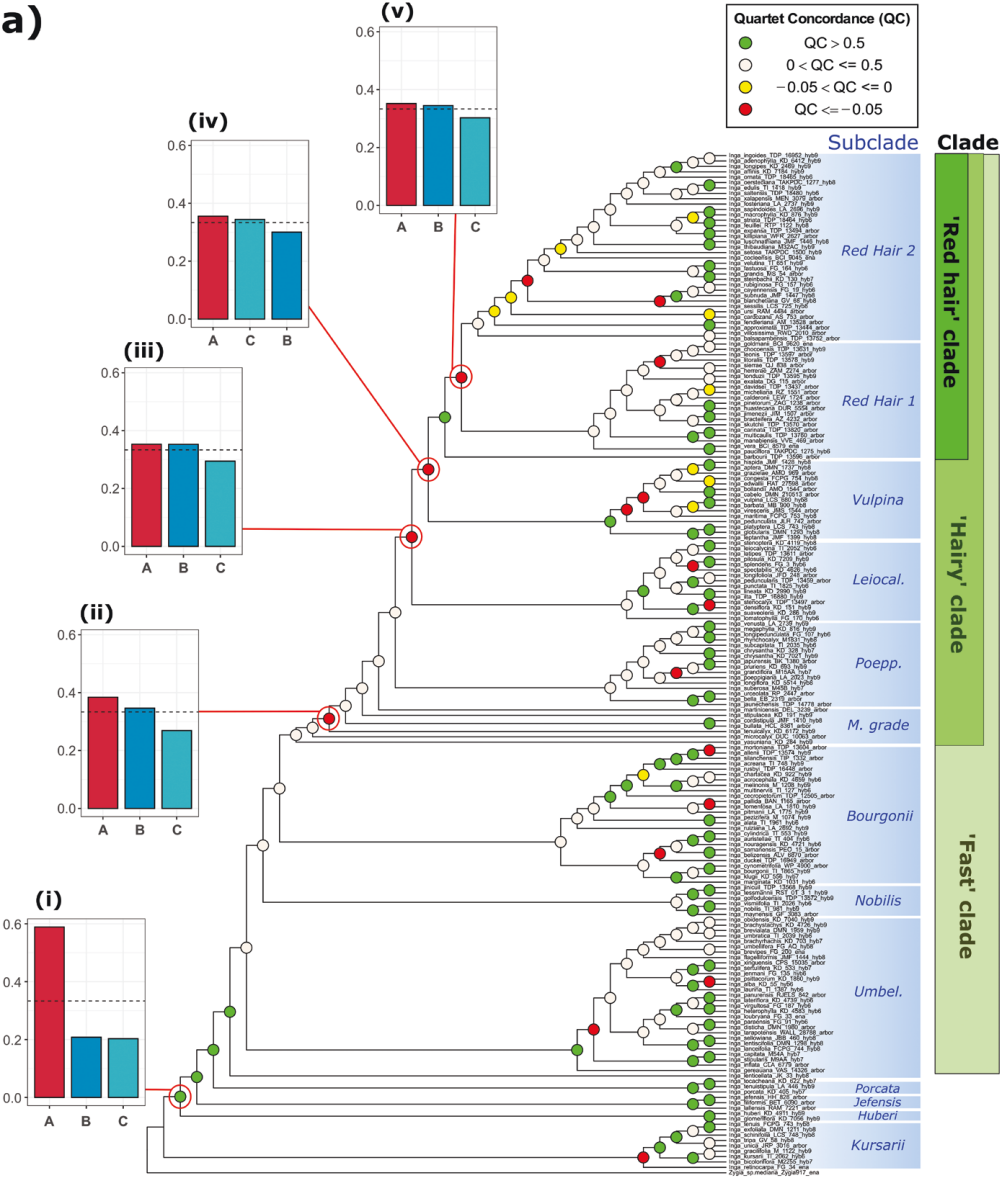

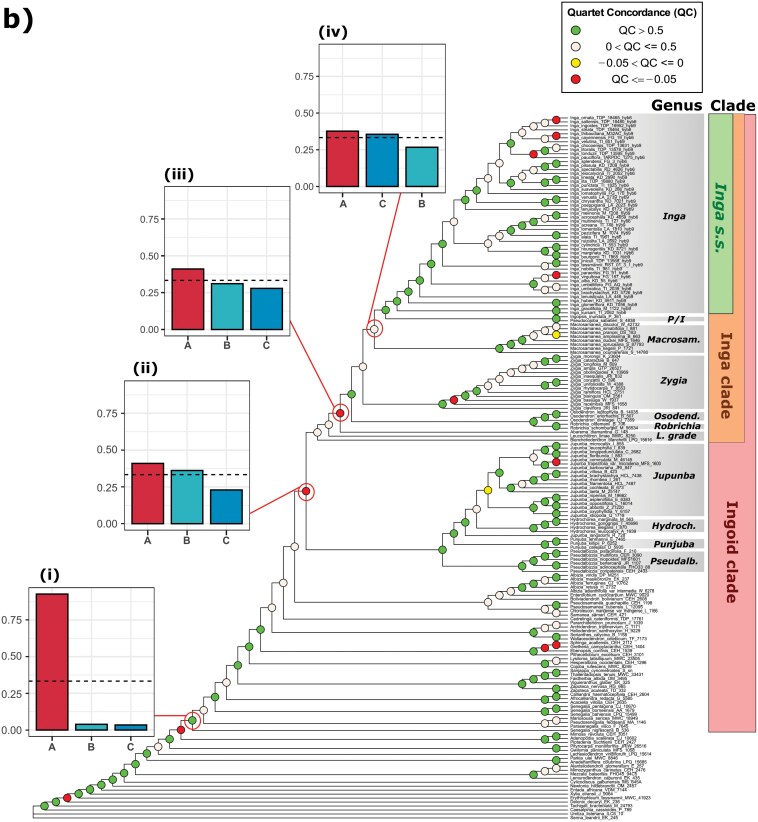
**a)**  *Inga* single-accession-per-species ASTRAL tree with QC values plotted on each node. Nodes of interest are additionally annotated with DiscoVista plots, showing the relative proportions of different discordant topologies. Quartet frequencies are represented as bar graphs, with red bars (left) representing the main topology from the ASTRAL analysis, and with blue and turquoise bars (middle and right) representing alternative topologies. Dashed horizontal lines mark the expectation for equal frequencies of the 3 possible topologies (Y = 0.333), i.e., maximal gene tree conflict. Node i indicates low proportions of both conflicting alternative topologies. Nodes ii–v indicate one major conflicting topology. Clades are annotated first by intrageneric subclade, and then with the broader clades within *Inga s.s.* in which they are nested (Redhair clade, Hairy clade, Fast clade). In shortened subclade annotations, “Leiocal.” = Leiocalycina subclade, “Poepp.” = Poeppigiana subclade, “M. grade” = Microcalyx grade, “Umbel.” = Umbellifera subclade. **b)**  *Inga* outgroup ASTRAL tree with QC values plotted on each node. Nodes of interest are additionally annotated with DiscoVista plots, showing the relative proportions of different discordant topologies. Quartet frequencies are represented as bar graphs, with red bars (left) representing the main topology from the ASTRAL analysis, and with blue and turquoise bars (middle and right) representing alternative topologies. Dashed horizontal lines mark the expectation for equal frequencies of the 3 possible topologies (Y = 0.333), i.e., maximal gene tree conflict. Node i indicates low proportions of both conflicting alternative topologies. Nodes ii–iv indicate one major conflicting topology. Clades are annotated by genus, and then by the broader phylogenetic clades in which they are nested (Inga clade, Ingoid clade). In shortened genus annotations, P/I = *Pseudocojoba/Ingopsis*, *Macrosam. *= *Macrosamanea*, *Osodend. = Osodendron*, *L. grade = Leucochloron* grade, *Hydroch. *= *Hydrochorea*, *Pseudoalb. = Pseudoalbizia*.

Our ASTRAL analyses indicated that most bipartitions were well supported across the 3 data sets, with local posterior probabilities (LPPs) >0.8 (Supplementary Fig. S3ai,b,c) and quartet scores of 0.47, 0.69, and 1.36 for the single-accession-per-species, PPD, and Outgroup data sets, respectively. Within *Inga*, we inferred 3 major nested clades (“Fast,” “Hairy,” and “Red Hair”) and 13 subclades within those (Fig. 1a). Interestingly, the concatenated IQ-TREE analysis of *Inga* recovered a nearly identical topology to the ASTRAL tree with high support (bootstrap (BS) > 90, Supplementary Fig. S3aii), but displayed a different branching order of the Vulpina, Leiocalycina, and Poeppigiana subclades. Paralog removal and trimming of hypervariable regions with PPD did not materially influence the resolution or topology of the ASTRAL *Inga* tree (Supplementary Fig. S3c). Within the outgroup tree, most generic splits are well supported (LPP > 0.9), and *Inga* was monophyletic (Supplementary Fig. S3b).

Genetic variation visualized with SplitsTree showed many shared splits within *Inga*, *Zygia*, and *Macrosamanea*, as well as between genera (Supplementary Fig. S4a,b). Shared splits are particularly evident within the “Hairy” clade of *Inga*, along with the “Red hair” clade nested within it (Supplementary Fig. S4a). SplitsTree analysis of the Outgroup data set also showed a number of shared splits within and between closely related Inga clade genera (e.g., *Macrosamanea*, *Ingopsis/Pseudocojoba*, *Robrichia*, and *Osodendron*; Supplementary Fig. S4b). Paralog removal and hypervariable site trimming with PPD resulted in a different clustering of *Inga* species, with the long branch leading to *Inga gereauana* bisecting the Red hair and Hairy clades (Supplementary Fig. S4c).

### Incongruence Is Common Within and Between Genera

Our analyses recovered phylogenetic incongruence both within and between Ingoid clade genera (Fig. 1a,b). Within *Inga*, negative QC and low QD scores, alongside DiscoVista, suggested a single conflicting topology was disproportionately represented within the Microcalyx grade, Leiocalycina, Vulpina, and Red hair subclades (QC in Fig. 1a nodes ii–v; QD in Supplementary Fig. S6a). However, most nodes in the *Inga* ASTRAL tree showed multiple conflicting topologies in similar proportions (i.e., QC scores between 0 and 0.5; Fig. 1a). Paralog removal and trimming with PPD had minimal effect overall, but led to slightly higher QC and QD scores at some nodes (QC: Supplementary Fig. S5; QD: Supplementary Fig. S6c). QI scores indicated all nodes were informative.

**Figure 2.**
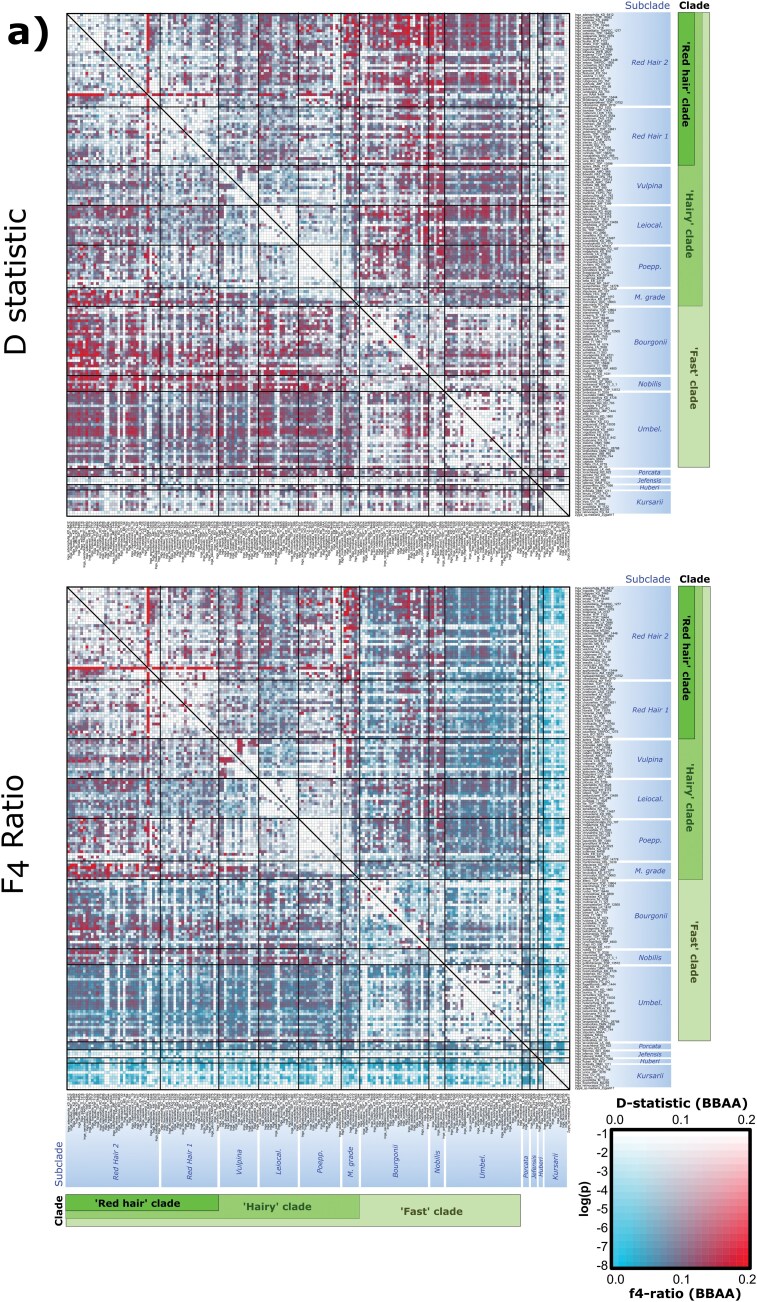

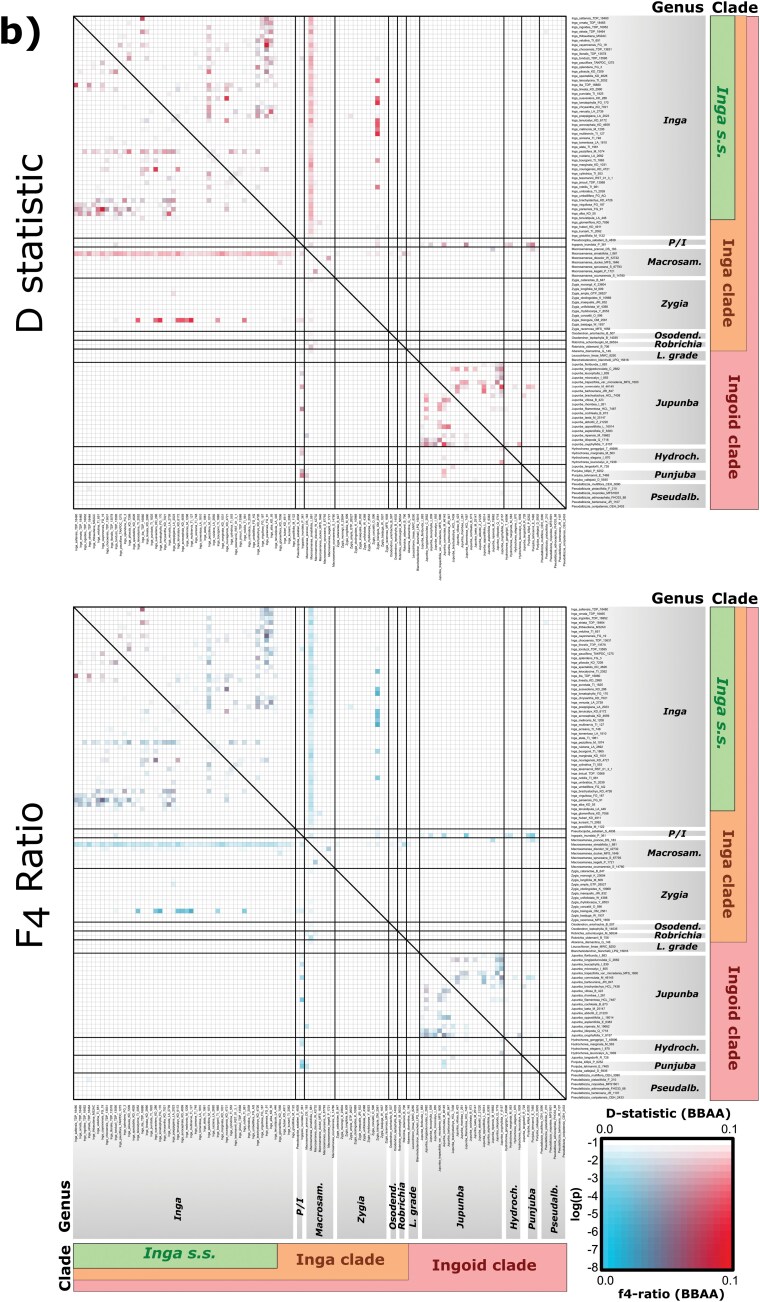
**a)** Heatmaps of per-triplet *D*-statistic (*D*, above) and *F*_4_ ratios (below) plotted for the single-accession-per-species *Inga* data set. Taxa P2 and P3 are displayed on the *x-* and *y*-axes in the same order as in Figure 1a. The color of each square signifies the *D-*statistic or *F*_4_ ratio estimate (blue = low estimate; red = high estimate). The saturation of these colors represents the significance for that test (see log(P) in inset box, bottom right). Clades are marked in the same colors as Figure 1a, representing subclades within *Inga* as well as the broader “Fast,” “Hairy,” and “Red hair” clades of *Inga*. *D* and *F*_4_ ratio estimates were made from species trios, ordered so that the P1 and P2 taxa possess the derived allele (the BBAA pattern) more frequently than the discordant ABBA and BABA patterns. This was to ensure that the P1/P2 taxa are more closely related to each other than to the P3 taxon and outgroup, as assumed by *D*-statistics. Clades are annotated first by intrageneric subclade, and then with the broader clades within *Inga s.s.* in which they are nested (Redhair clade, Hairy clade, Fast clade). In shortened subclade annotations, “Leiocal.” = Leiocalycina subclade, “Poepp.” = Poeppigiana subclade, “M. grade” = Microcalyx grade, “Umbel.” = Umbellifera subclade. **b)** Heatmaps of minimum per-triplet *D*-statistic (*D*, above) and *F*_4_ ratios (below) plotted for the Outgroup data set. Taxa P2 and P3 are displayed on the *x-* and *y*-axes in the same order as in Figure 1b. The color of each square signifies the *D-*statistic or *F*_4_ ratio estimate (blue = low estimate; red = high estimate). The saturation of these colors represents the significance for that test (see log(P) in inset box, bottom right). Clades are marked in the same colors as Figure 1b, representing different genera and the “Ingoid clade,” “Inga clade,” and the genus *Inga s.s. D* and *F*_4_ ratio estimates were made from species trios, ordered so that the P1 and P2 taxa possess the derived allele (the BBAA pattern) more frequently than the discordant ABBA and BABA patterns. This was to ensure that the P1/P2 taxa are more closely related to each other than to the P3 taxon and outgroup, as assumed by *D*-statistics. Clades are annotated by genus, and then by the broader phylogenetic clades in which they are nested (Inga clade, Ingoid clade). In shortened genus annotations, P/I = *Pseudocojoba/Ingopsis*, *Macrosam. *= *Macrosamanea*, *Osodend. = Osodendron*, *L. grade = Leucochloron* grade, *Hydroch. *= *Hydrochorea*, *Pseudoalb. = Pseudoalbizia*.

Most nodes in the outgroup tree recovered higher QC scores, indicative of more phylogenetic concordance (Fig. 1b). This was with the exception of a few nodes involving *Zygia*, *Macrosamanea*, *Abarema*, and the clade containing *Jupunba*, that had negative QC scores (Fig. 2a nodes ii–iv; QD trees in Supplementary Fig. S6b) or showed multiple conflicting topologies (QC between 0 and 0.5). For both trees, the deepest divergences had more concordant quartet topologies (Fig. 1a node i; Fig. 1b node i).

### Reticulation Occurs at Multiple Phylogenetic Scales

The overrepresentation of one incongruent topology that we inferred for several nodes (Fig. 1aii–v; Fig. 1bii–iv) was reinforced by the high *D*-statistics, *F*_4_ ratios, and *F*_branch_ scores that we calculated for all 3 data sets. This suggests that reticulation contributed to incongruence at these nodes.

Within *Inga*, significant *D*-statistics up to 0.2 were observed most frequently between the “Red hair” clade and the Bourgonii/Nobilis subclades (Fig. 2a). More broadly, we found many significant *D*-statistic results across the *Inga* tree, despite our stringent treatment for multiple testing and ABBA-clustering. “Red hair” clade species shared up to 20% of their sequence variation with the Microcalyx grade and the Vulpina/Leiocalycina/Poeppigiana subclades (*F*_4_ ratio = 0.2, *P* < 0.01). *F*_4_ ratios also strongly suggested introgression events within the Red Hair clade (involving *Inga ursi*) and Vulpina subclade (involving *I. hispida/I. barbata*). Removal of putative paralogs with PPD did not materially influence the *F-* or *D*-statistics we inferred (Supplementary Fig. S7). *F*_branch_ also showed ca. 20% excess allele sharing “Red hair” clade species (e.g., *Inga pauciflora*, *I. ursi*) and the Vulpina, Poeppigiana, Bourgonii, and Microcalyx grade subclades (Supplementary Fig. S8a).

In the broader Outgroup data set, many *D*-statistic tests were filtered out due to insignificant clustering of ABBA patterns (Fig. 2b). However, *D*-statistics suggested some introgression in other Ingoid clade genera (*Macrosamanea* and *Jupunba*) as well as between *Zygia/Macrosamanea* and *Inga* (*D =* 0.1; Fig. 2b). *F*_4_ ratio and *F*_branch_ scores recovered more limited evidence of introgression, with the highest scores occurring within closely related species pairs in *Inga* and *Jupunba* (Supplementary Fig. S8b).

**Figure 3.**
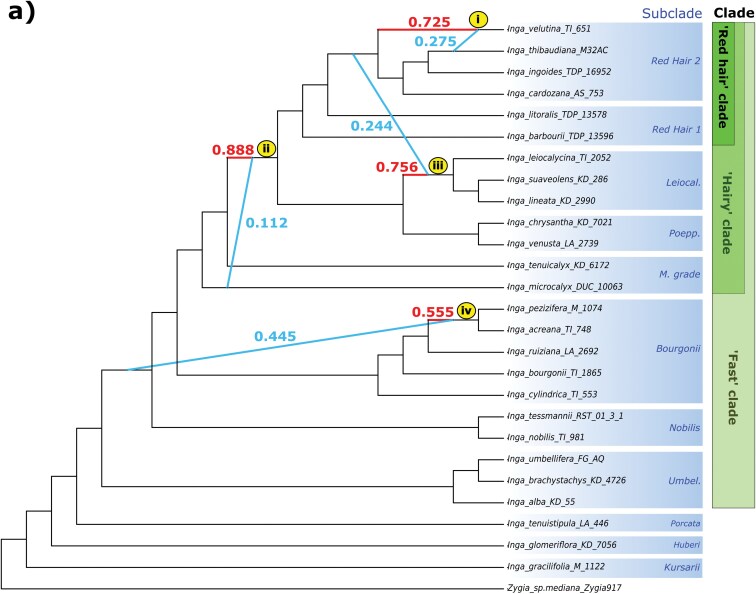

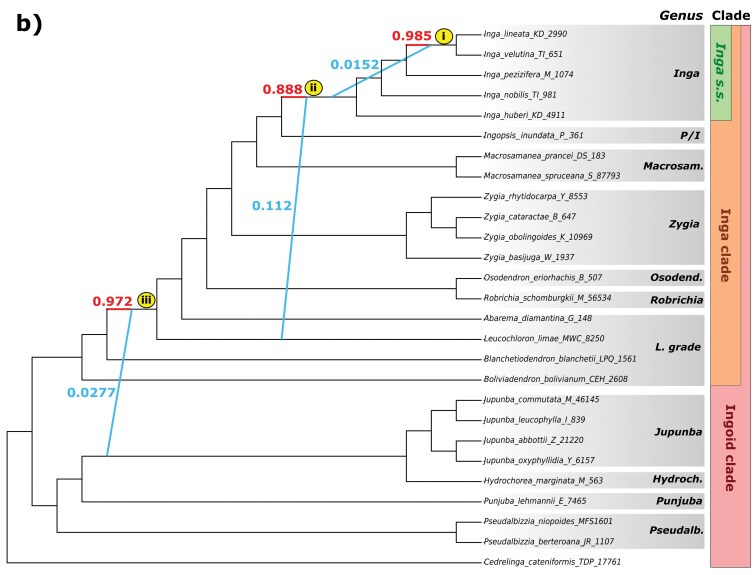
a) Phylogenetic network with 4 reticulation events (*hmax =* 4; i–iv), estimated using *SNaQ!* in the Julia package PhyloNetworks based on the “Single-accession-per-species” *Inga* data set. Emboldened, coloured branches (blue and red) indicate inferred hybridization events, and numbers next to branches indicate inheritance probability (γ), roughly equal to the proportion of genetic variation contributed by each lineage to a reticulation event. Clades are annotated first by intrageneric subclade, and then with the broader clades within *Inga s.s.* in which they are nested (Redhair clade, Hairy clade, Fast clade). In shortened subclade annotations, “Leiocal.” = Leiocalycina subclade, “Poepp.” = Poeppigiana subclade, “M. grade” = Microcalyx grade, “Umbel.” = Umbellifera subclade. b) Phylogenetic network with 3 reticulation events (*hmax =* 3; i–iii), estimated using *SNaQ!* in the Julia package PhyloNetworks based on the “Outgroup” data set. Emboldened, coloured branches (blue and red) indicate inferred hybridization events and numbers next to branches indicate inheritance probability (γ), roughly equal to the proportion of genetic variation contributed by each lineage to a reticulation event. Clades are annotated by genus, and then by the broader phylogenetic clades in which they are nested (Inga clade, Ingoid clade). In shortened genus annotations, P/I = *Pseudocojoba/Ingopsis*, *Macrosam. *= *Macrosamanea*, *Osodend. = Osodendron*, *L. grade = Leucochloron* grade, *Hydroch. *= *Hydrochorea*, *Pseudoalb. = Pseudoalbizia*.

Our PhyloNetworks analyses suggested that 4 reticulation events best fit the observed quartet concordance factors within *Inga* (−loglikelihood *hmax* = 4, Supplementary Fig. S9ai; Supplementary Table S4). We inferred reticulation firstly within the “Red hair 2” subclade, between the *Inga velutina* and *I. thibaudiana* lineages, with inheritance probabilities (γ) suggesting the *I. thibaudiana* lineage contributed ca. 27% of *I. velutina*’s genetic material (Fig. 3ai; γ = 0.275). The other 3 reticulation events occurred deeper in the tree, from the *I. microcalyx* lineage into the base of the Poeppigiana/Leiocalycina/Redhair subclades (Fig. 3aii; γ = 0.112), from the Red Hair 2 subclade into the Leiocalycina subclade (Fig. 3aiii; γ = 0.244) and from the Umbellifera subclade/Fast clade split into the Bourgonii subclade (Fig. 3aiv; γ = 0.445).

Within *Inga* subclades, we inferred the most reticulation events within the Bourgonii subclade (*hmax* = 4) and the fewest within the Vulpina subclade (*hmax = *1), with all other subclades recovering 2 reticulation events (Supplementary Fig. S9aii and iii). Many of these within-subclade events involve the same taxa with reticulate histories inferred using *D*-statistics, *F*_4_ ratios, and genus-level PhyloNetworks analyses (e.g., *Inga microcalyx*, *I. balsapambensis*, *I. hispida*).

Among Ingoid clade “Outgroup” species, we inferred 3 reticulation events (−loglikelihood *hmax* = 3, Supplementary Fig. S9b; Supplementary Table S4). We firstly recovered reticulation from the base of *Inga* into members of the Red Hair 2 and Leiocalycina clades (Fig. 3bi; γ = 0.0152). We also inferred reticulation from the *Leucochloron limae* lineage into *Inga* (Fig. 3bii; γ = 0.112) and from more distantly related Ingoid clade lineages (*Jupunba/Hydrochorea*) into the lineage leading to *L. limae* and the rest of the Inga clade (Fig. 3biii; γ = 0.0277).

### Levels of Introgression and Selection Differ Between Loci in Inga

Our estimates of introgression and selection varied widely across the target capture loci we analyzed. Per-locus introgression (*f*  _*dM*_) varied between 0.0001 and 0.664 across all 3 subclade subsets, approaching the maximum *f*  _*dM*_ score of 1 in some subsets (Table 1; Supplementary Fig. S10). The “Bourgonii + Microcalyx Grade + Red Hair” subset produced the highest *f*  _*dM*_ scores overall (Table 1). Across the analyses, the highest *f*  _*dM*_ scores were observed in the single-copy phylogenetically informative loci, which were the most numerous (Supplementary Fig. S10).

**Table 1.** Summaries of per-locus *f*  _dM_ statistics across data subsets. Columns, from left to right, indicate the subclade data subset that *f*  _*dM*_ was estimated for across all loci, the number of species in each subset, the number of loci in each subset, and the minimum, maximum, and mean *f*  _*dM*_ scores for each subset. The phylogenetic position of the subclade data subsets that were used for running the *f*  _*dM*_ analyses listed in the leftmost column are illustrated in Supplementary Figure S1. In the first column, “Leio.” = Leiocalycina subclade, “Vulp.” = Vulpina subclade, “R.H.” = Red Hair clade (i.e., Red Hair 1 + 2 subclades), “Mic.” = Microcalyx grade, “Bour.” = Bourgonii subclade. In the final column, the number (in parentheses) and percentage of all loci inferred to be under selection (i.e., with FDR-corrected BUSTED *P*-value < 0.05) is shown for each subset.

Summaries of per-locus *f* _dM_ statistics across data subsets						
Data subset	*N* species	*N* loci	Min *f* _*dM*_	Max *f* _*dM*_	Mean *f* _*dM*_	% loci under selection
Leio. + Vulp. + R.H.	87	889	0.0001	0.581	0.14	66.591% (592)
Mic. + Leio. + R.H.	79	874	0.0003	0.662	0.09	61.899% (541)
Bour. + Mic. + R.H.	90	875	0.0002	0.664	0.107	68.342% (598)

In total, BUSTED inferred evidence of positive selection in between 61% and 68% of analyzed loci per subset after multiple-testing correction (Table 1). Across all 3 subclade subsets, more “defence chemistry” loci showed evidence of selection than the null expectation, whereas the opposite was true for other locus annotation classes (Supplementary Fig. S11). However, χ^2^ tests only showed a significant association between selection result and locus annotation in the “Bourgonii + Microcalyx grade + Red hair” subset (χ^2^ = 10.036, df = 3, *N* = 875, *P* = 0.0182) (Supplementary Table S5).

Our ANCOVA analyses showed significant differences in *f*  _*dM*_ score means between locus annotations in the “Leiocalycina + Vulpina + Red hair” subset (*P* = 0.0003, *F*(3,873)* = *6.24, η^2^ = 0.02), with “defence chemistry” and “differentially expressed” loci showing elevated *f*  _*dM*_ scores in loci under selection, although these were not significant (Supplementary Table S6, Supplementary Fig. S10). However, ANCOVA did reveal significant differences in *f*  _*dM*_ means between locus annotations when loci experienced selection in the “Bourgonii + Microcalyx grade + Red hair” subset (*P* = 0.0461, *F*(3,859)* =* 5.77, η^2^ = 0.009) (Supplementary Table S6, Supplementary Fig. S10).

## Discussion

### *Diversification of* Inga *and the Ingoid Clade*

Our analyses recovered well-supported phylogenetic trees for *Inga* as well as the broader Ingoid clade within which it is nested (LPP > 0.8, BS > 90; Supplementary Fig. S3ai and ii,b,c). The phylogenetic tree of *Inga* we inferred marks a great advance in the resolution of interspecies relationships when compared to previous phylogenetic work using fewer loci and species (e.g., Richardson et al. 2001; Kursar et al. 2009; Dexter et al. 2010; Nicholls et al. 2015). Thus, our phylogenetic tree provides the best available framework to investigate the role of hybridization in this species-rich group.

Our analyses revealed 3 nested clades within *Inga* (Fig. 1a) subdivided into 12 subclades and 1 grade. The deepest-level group is the “fast” clade, at the base of which there was a substitution rate shift inferred by other studies (Ringelberg et al. 2023). Nested within the “Fast” clade is the “Hairy” clade, in which many species possess indumentum (hair-like trichomes) on young leaves as a defense against herbivores (Agrawal 1999; Coley et al. 2018). Finally, nested within the “Hairy” clade is the “Red hair” clade, containing species that possess both dense indumentum and diverse defence chemistry (Coley et al. 2018). SplitsTree inferred a high degree of shared genetic variation within the “Hairy” and ‘Red hair’ clades of *Inga*, as well as within and between other Inga clade genera (*Macrosamanea*, *Zygia*, *Ingopsis*, and *Pseudocojoba*) (Supplementary Fig. S4a,b). These closely related species, some of which were formerly in the genus *Zygia*, have a history of non-monophyly as described by Ferm et al. (2019, 2024), and further cases of generic non-monophyly within the Ingoid clade have been highlighted by Ringelberg et al. (2022).

### Phylogenetic Incongruence Is Widespread and Influenced by Introgression

We found phylogenetic incongruence both within and between Ingoid clade genera using QC scores and DiscoVista (Fig. 1a,b). For the Singlesp and Outgroup data sets, we found one conflicting topology overrepresented at several incongruent nodes (Fig. 1a nodes ii–v; Fig. 1b nodes ii–iv; see also QD trees in Supplementary Fig. S6a,b), suggesting reticulation (Wendel and Doyle 1998). This discordance was particularly evident within the Microcalyx grade, and Leiocalycina, Vulpina, and Red hair subclades (Fig. 1a nodes ii–iv), subclades that also differed in branching order between the ASTRAL and concatenated IQ-TREE analyses (Supplementary Fig. S3ai–ii). Both reticulation and ILS result in multiple evolutionary histories across the genome, and so averaging across these histories by concatenating loci often results in spurious phylogenetic relationships, explaining this difference in branching order (Degnan and Rosenberg 2009). Many deeper nodes across Figure 1a and 1b also showed several conflicting topologies at similar frequencies (QC scores between 0 and 0.5) indicating ILS, which is common in rapidly diversifying clades like *Inga* (Degnan and Rosenberg 2009).

However, our explicit tests for introgression using *D*-statistic, *F*_4_ ratios, and *F*_branch_ (Fig. 2a,b; Supplementary Fig. S8a,b) also inferred widespread introgression across *Inga*. Our *F*_4_ ratio estimates suggested that up to 20% of genetic variation was shared between some *Inga* species. This result was corroborated by the stringent *F*_branch_ analysis which is less prone to inferring spurious introgression, since it averages *F*-statistics across related branches (Supplementary Fig. S8a). Interestingly, this is a similar proportion of shared variation as reported in radiations catalyzed by “ancient” hybridization (e.g., Lake Victoria cichlid fish (Meier et al. 2017)). We also recovered limited evidence of reticulation in other Ingoid clade genera (e.g., *Jupunba*, *Zygia* into *Inga*) likely because most introgression signal could not be distinguished from homoplasy after stringent filtering by our ABBA-clustering analysis.

PhyloNetworks inferred at least 4 migration events in *Inga* (Fig. 3a), involving several subclades with pervasive evidence of introgression in our *D*- and *F*-statistics (Fig. 2a). Notably, Figure 3a (node i) captures signal of the introgression events we inferred in the Red hair 2 subclade using *D*- and *F*_4_-statistics (Fig. 2a). Similarly, nodes ii and iii of Figure 3a reflect the introgression we inferred between the Microcalyx grade and the Leiocalycina, Vulpina, and Red hair subclades in our *D*- and *F*-statistics. Finally, the strong introgression signal we inferred in the Bourgonii and Nobilis subclades with *D*- and *F*-statistics (Fig. 2a) was recovered in our PhyloNetworks analysis (Fig. 3a, node iv), involving deep reticulation within *Inga*. We also inferred at least 3 migration events in the Outgroup data set, which involved *Inga* (Fig. 3b node i–ii), the *Leucochloron* grade (node ii–iii), and *Jupunba* (node iii). While *D*- and *F*_4_-statistics recovered most evidence of introgression in the Outgroup data set within *Inga* and *Jupunba* (Fig. 2b), these were only broadly reflective of the PhyloNetworks analyses due to the stringent filtering of *D*- and *F*-statistic comparisons that we performed using ABBA-clustering.

The widespread introgression we inferred between non-sister species throughout the radiation of *Inga* may be congruent with the syngameon hypothesis of adaptive radiation (Seehausen 2004; see also Wogan et al. 2023), rather than a “hybrid swarm” preceding the radiation that catalyzed diversification. Periodical hybridization within a syngameon may be adaptive for Amazonian tree species, which are typified by large, dispersed populations (ter Steege et al. 2013). Periodical hybridization can elevate genetic diversity and prevent Allee effects, such as inbreeding, at low population densities (Cannon and Lerdau 2015, 2019). *Inga* species are highly dispersible, with the entirety of Amazonia acting as a species pool for the assembly of local *Inga* communities (Dexter et al. 2017). This may facilitate introgression between *Inga* species, particularly given *Inga*’s generalist pollination syndrome and overlapping phenology (Koptur 1983). Recent work on Amazonian trees has also documented putative local syngameons in other genera, for example, between *Brownea* species (Schley et al. 2020) and among 3 *Eschweilera* species in Brazil (Larson et al. 2021).

Further evidence for the syngameon radiation hypothesis is the introgression we inferred across whole subclades using *D*- and *F*-statistics (Fig. 2a,b; Supplementary Fig. S8a,b). Shared variation spanning subclades would be expected following introgression within ancestral syngameons, as introgressant variants are inherited by descendent species (e.g., Meier et al. 2017; Meyer et al. 2017; Schley et al. 2020). While such a pattern might result from the violation of assumptions made by *D*- and *F*-statistics (e.g., that there is no substitution rate variation (Patterson et al. 2012)), we were extremely careful to account for this by using the newly implemented “ABBA-clustering” tool in Dsuite (Koppetsch et al. 2024). This tool tests for significant clustering of ABBA site patterns (which would be expected following introgression) to distinguish them from homoplasy (in which case ABBA patterns would be more dispersed throughout the genome).

We also inferred introgression at the base of several clades with PhyloNetworks (e.g., Fig. 3a, node iv; Fig. 3b, node ii), again reflecting the inheritance of introgressed loci by descendent species after “ancient” hybridization. PhyloNetworks is not prone to biases caused by substitution rate variation (Koppetsch et al. 2024), and yet still recovered introgression events that reflected our *D*- and *F-*statistic results. Worth noting, however, is that such events may also reflect introgression with “ghost” lineages that were not sampled, or have gone extinct since the introgression event (Tricou et al. 2022). This suggests introgression may be more widespread throughout *Inga*’s history than we were able to infer with our current sampling.

In all, *Inga* may be representative of other large tree genera in Neotropical rainforests, which account for half of Amazonian tree diversity. Large genera such as *Protium* and *Eschweilera* also show high sympatry in local communities (Bermingham and Dick 2001), alongside emerging evidence of introgression (Larson et al., 2021; *Eschweilera*), much like *Inga*. Assuming *Inga* is representative of these other groups, our analyses suggest that introgression is more widespread than previously thought in species-rich Amazonian tree genera (Ashton 1969).

However, the retention of ancestral polymorphisms in descendent lineages (known as ILS (Doyle 1992)), is also pervasive in rapid Amazonian tree radiations. This was shown by our results (Figs. 1a,b and 2a,b; Supplementary Fig. 6a,b) and has been demonstrated extensively in the Mimosoid legumes, to which the Ingoid clade belongs (Koenen et al. 2020). ILS arises in these groups because the probability of coalescence (i.e. sorting of derived alleles into descendent lineages reflecting speciation history) in *t* generations decreases with increasing effective population size (Fisher 1930; Wright 1931; Kingman 2000). Most rainforest trees have large, widespread populations (ter Steege et al. 2013), such that genome-wide coalescence and sorting of alleles may be unlikely to have yet occurred. This is particularly true of 'young', rapidly diversifying rainforest tree genera such as *Inga* (discussed in Pennington R. T. and Lavin 2016).

### *Introgression and Selection Influence the Evolution of Defense Chemistry Loci in* Inga

We detected multiple deep introgression events across *Inga*, suggesting that some loci transferred by introgression are retained over time. It is likely that these loci were not immediately deleterious and were not subject to purifying selection, perhaps residing in areas of the genome that are distant from incompatibility loci, allowing them to recombine freely (Edelman et al. 2019). It is also possible that these regions are adaptive and so are maintained by positive selection, as shown in temperate tree species (e.g., Rendón-Anaya et al. 2021). This is particularly interesting in the context of chemical defenses against insect herbivores, since these defenses are critical for survival, coexistence, and ecological divergence in *Inga* (Kursar et al. 2009; Coley et al. 2018; Forrister et al. 2023).

We found differences in the proportion of loci under selection between locus annotation classes, with elevated numbers of defence chemistry loci under selection (Supplementary Fig. S11), particularly in the “Bourgonii + Microcalyx grade + Red hair” subset (χ^2^ = 10.036, *N* = 875, df = 3, *P*-value = 0.0186) (Supplementary Table S5). Previous work using phylogenetic comparative methods demonstrated divergent evolution in defense chemical profiles among sister species of *Inga* (Forrister et al. 2023). Our results suggest a potential mechanism underlying this divergent evolution, given that we recovered molecular evidence of positive selection in defense chemistry loci. This provides an important exemplar for understanding the assembly of diverse rainforest tree communities—herbivore pressure structures tree communities, and so divergent defense chemistry facilitates ecological coexistence in speciose rainforest tree genera like *Inga* (Kursar et al. 2009; Forrister et al. 2019).

Our estimates of per-locus introgression (*f*  _*dM*_) varied widely across the loci we analyzed and across subclade subsets (Table 1; Supplementary Fig. S10). Locus annotation best explained variance in introgression (*f*  _*dM*_) across loci in the “Leiocalcycina + Vulpina + Red hair” subset (*P* = 0.0003, *F*(3,873)* = *6.245, η^2^ = 0.02), with slightly higher mean introgression for defense loci under selection (Supplementary Fig. S10). Similarly, for the “Bourgonii + Microcalyx grade + Red hair” subset, both locus annotation and selection result best explained *f*  _*dM*_ variation (*P* = 0.0461, *F*(3,859) = 5.77, η^2^ = 0.009) (Supplementary Fig. S10; Supplementary Table S6) but with a relatively low effect size. *f*  _*dM*_ scores were marginally higher in defense chemistry loci that were under selection in some subsets (Supplementary Fig. S10), suggesting a plausible role of introgression in generating adaptive defense chemistry phenotypes in *Inga*. Moreover, novel defense chemicals in *Inga* likely arise through the combination of chemical precursors, rather than de novo innovation (Coley et al. 2018). This might suggest a role for admixture in generating defense chemistry, rather than solely selective mechanisms at the single-gene level (e.g., negative frequency-dependent selection retaining rare polymorphisms (Wright 1939)). Thus, novel combinations of defenses resulting from introgression may confer resistance to different herbivore communities. This in turn could facilitate colonization of, and adaptation to, new areas with different suites of herbivores, in a similar way to   how introgression of wing pattern genes facilitates adaptation to local mimicry rings in *Heliconius* butterflies (The Heliconius Genome Consortium 2012). While a small proportion of elevated *f*  _*dM*_ scores may result from ILS, where a local genealogical tree in a window resembles a tree expected under introgression by chance, we averaged all per-window *f*  _*dM*_ scores across loci to reduce the impact of such outliers.

In light of the Janzen–Connell hypothesis, where higher densities of conspecifics with the same defenses leads to increased mortality from herbivores (Janzen 1970; Connell 1971), possession of a rare, introgressant defense chemistry phenotype is likely to be adaptive, as fewer herbivores in the new area can overcome it. Adaptive introgression that facilitates colonization of new habitats is well known in plants (Suarez‐Gonzalez et al. 2016), particularly in the context of defense against herbivores (Whitney et al. 2006), and may have influenced the rapid radiation of *Inga*.

## Conclusions

Our analyses indicate that rapid Amazonian tree radiations (e.g., *Inga*) display evidence of introgression, in addition to ILS. The introgression we inferred may be evidence of “syngameons” of co-occurring interfertile species, which are created by dispersal-assembled local tree communities in Neotropical rainforests. This introgression may have influenced adaptation throughout the *Inga* radiation by transferring adaptive loci between speciating lineages. Specifically, we found that loci relating to defense chemistry show more evidence of selection than expected by chance, and those loci under selection have slightly higher proportions of introgression. This suggests that introgression may facilitate adaptation, local coexistence, and diversification in Amazonian trees.

## Supplementary Material

Data available from the Dryad Digital Repository: https://dx.doi.org/10.5061/dryad.tb2rbp09n.

## Data Availability

Data, including all sequence alignments, phylogenetic trees, and vcf files, are available from the Dryad Digital Repository: https://doi.org/10.5061/dryad.tb2rbp09n. The accession numbers for all data collated from previous studies and those newly submitted for this study are found in Supplementary Table S1. All nucleotide sequence data produced by this study are available on the NCBI Sequence Read Archive under the study accession PRJEB84192.
